# The hyperbolic geometry of financial networks

**DOI:** 10.1038/s41598-021-83328-4

**Published:** 2021-02-26

**Authors:** Martin Keller-Ressel, Stephanie Nargang

**Affiliations:** Institute for Mathematical Stochastics, TU Dresden, 01062 Dresden, Germany

**Keywords:** Applied mathematics, Scientific data

## Abstract

Based on data from the European banking stress tests of 2014, 2016 and the transparency exercise of 2018 we construct networks of European banks and demonstrate that the latent geometry of these financial networks can be well-represented by geometry of negative curvature, i.e., by hyperbolic geometry. Using two different hyperbolic embedding methods, hydra+ and Mercator, this allows us to connect the network structure to the popularity-vs-similarity model of Papdopoulos et al., which is based on the Poincaré disc model of hyperbolic geometry. We show that the latent dimensions of ‘popularity’ and ‘similarity’ in this model are strongly associated to systemic importance and to geographic subdivisions of the banking system, independent of the embedding method that is used. In a longitudinal analysis over the time span from 2014 to 2018 we find that the systemic importance of individual banks has remained rather stable, while the peripheral community structure exhibits more (but still moderate) variability. Based on our analysis we argue that embeddings into hyperbolic geometry can be used to monitor structural change in financial networks and are able to distinguish between changes in systemic relevance and other (peripheral) structural changes.

## Introduction

Network models based on hyperbolic geometry have been successful in explaining the structural features of informational^1^, social^2^ and biological networks^3^. Such models provide a mathematical framework to resolve the conflicting paradigms of preferential attachment (attraction to *popular* nodes) and community effects (attraction to *similar* nodes) in networks^4–6^.

Just as the geometric structure of a social network determines the diffusion of news, rumors or infective diseases between individuals^7^, the geometric structure of a financial network influences the diffusion of financial stress between financial institutions, such as banks^8–11^. Indeed, the lack of understanding for risks originating from the systemic interaction of financial institutions has been identified as a major contributing factor to the global financial crisis of 2008^12^. While many recent studies have analysed the mechanisms of financial contagion in theoretical or simulation-based settings, less attention has been payed to the structural characteristics and the geometric representation of real financial networks. Although evidence of hyperbolic structure has been uncovered for international trade networks^13^, no such analysis has been carried out for networks of financial institutions. Identifying a suitable geometric representation for such networks can help to monitor and quantify structural change, and—in the case of hyperbolic geometry—even distinguish structural change in terms of systemic importance from changes in the network’s peripheral structure. Moreover, a geometric representation can form the basis of analytic models of contagion processes and their optimal control in future research.

Here, we consider financial networks inferred from bank balance sheet data, as collected and made available by the European Banking Authority (EBA) within the European banking stress test and transparency exercises of 2014, 2016 and 2018^14,15^. We show that these networks can be embedded into low-dimensional hyperbolic space with considerably smaller distortion than into Euclidean space of the same dimension, suggesting that the paradigm of latent hyperbolic geometry also applies to financial networks. In addition, we demonstrate that the hyperbolic geometric representation compares favorably to a degree-corrected stochastic block model^16^—a popular non-geometric network model. Furthermore—following Papadopoulos et al.^4^—we decompose the embedded hyperbolic coordinates into the dimensions of *popularity* and *similarity* and demonstrate that these dimensions align with *systemic importance* and membership in *regional banking clusters* respectively. Finally, the longitudinal structure of the data allows us to track changes in these dimensions over time, i.e., to track the stability of systemic importance and of the peripheral community structure over time.

## Results

### Inference of financial networks

Contagion in financial networks is a complex process, which can take place through several parallel (and potentially interacting) mechanisms and channels^17^. These mechanisms include direct bank-to-bank liabilities^18^, bank runs^19^, and market-mediated contagion through asset sales^17,20–22^ (‘fire-sale contagion’); see also (French et al.^12^, p. 21ff). Here, we focus on the channel of fire-sale contagion, which has been singled out—both in simulation^21^ and in empirical studies^20^—as a key mechanism of financial contagion. Moreover, the propensity of fire-sale contagion can be quantified from available balance sheet data, using liquidity-weighted portfolio overlap (LWPO)^22,23^ as an indicator (see “Methods” for details).

Our inference of financial networks follows a two-stage mechanism: First, we construct a weighted bipartite network in which banks $B = (b_1, \ldots , b_n)$ are linked to a common pool of assets $A = (a_1, \ldots , a_m)$, which consist of sovereign bonds classified by issuing country and by different levels of maturity. In the second step we perform a one-mode projection of this network on the node set *B*, using the LWPO of two banks $b_i, b_j \in B$ to determine the weight $w_{ij}$ of the link between the corresponding nodes. For any of the years $y \in \{2014, 2016, 2018\}$, the result is an undirected, weighted network $N_y$ of banks, in which two banks are connected if and only if they hold common assets. The link weight $w_{ij}$, normalized to [0, 1], represents the susceptibility of two banks $b_i, b_j$ to financial contagion, quantified by their LWPO.

### Network features

**Figure 1:**
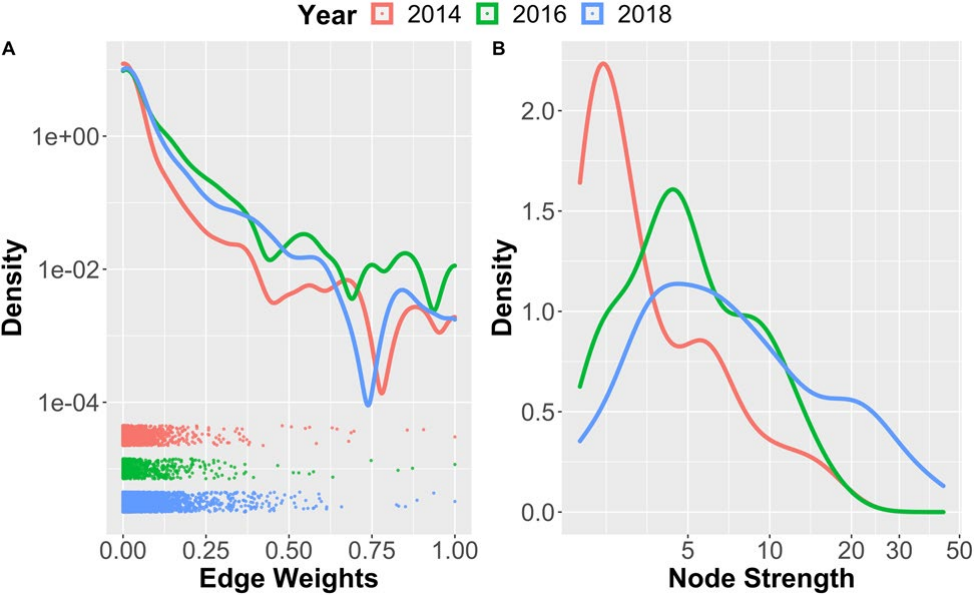
Densities of edge weights (**A**) and of node strengths (**B**) in the EBA financial networks of 2014, 2016 and 2018.

The inferred networks are very dense (densities: $\rho _{2014} = 0.86$, $\rho _{2016} = 0.96$, $\rho _{2018} = 1.00$),   i.e., almost all pairs of banks hold *some* common assets. However, the distribution of weights is highly skewed (see Fig. 1A), with most of the connections exhibiting very small weights. In other words, the networks are dominated by a ‘sparse backbone’ of a few strong connections, which represent the dominant channels of potential contagion of financial distress. The same skew is present in the distribution of node strengths (see Fig. 1B) with a few strong nodes dominating over a majority of weaker nodes in all years.

**Figure 2:**
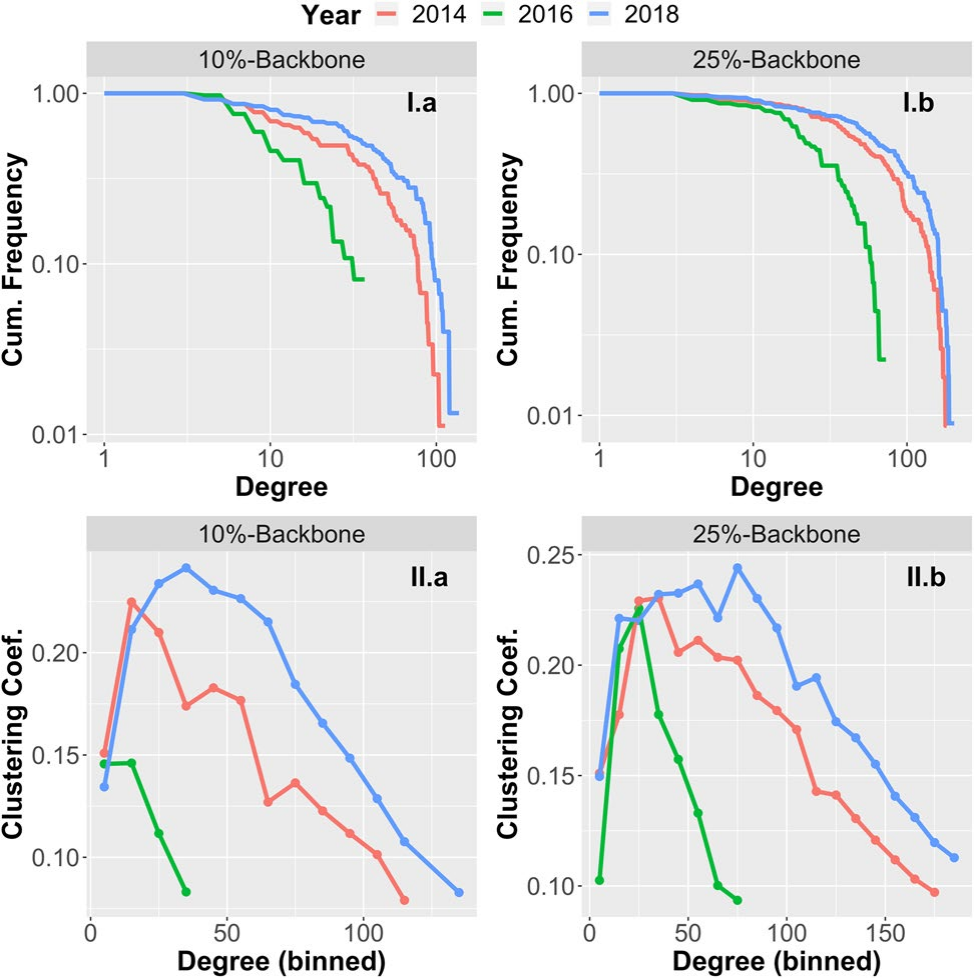
Degree distribution (**I**) and local clustering coefficients (**II**) in the 10%-backbone (**a**) and the 25%-backbone (**b**) of the EBA financial networks of 2014, 2016 and 2018.

To extract more salient connectivity information from these highly connected networks, we also consider the ‘$p\%$-Backbone’ of each network, formed by the upper $p\%$-quantile of highest-weighted edges (We have also considered disparity filtering^24^ as an alternative method for backbone extraction; see ‘Comparison of methods and robustness checks’ below.). Figure 2 shows the degree distribution and the local clustering coefficient (in dependence on node degree) for the 10%- and the 25%-Backbone. While there is no evidence of a scale-free degree distribution, the clustering coefficient displays an interesting pattern: It is highest for medium-degree nodes and then decreases with increasing node degree. This indicates that high-degree nodes (i.e. highly connected banks) typically act as hubs between lower-degree nodes (i.e. ‘normal’ banks) without a direct link.

### Latent network geometry

#### Network representation methods

**Figure 3:**
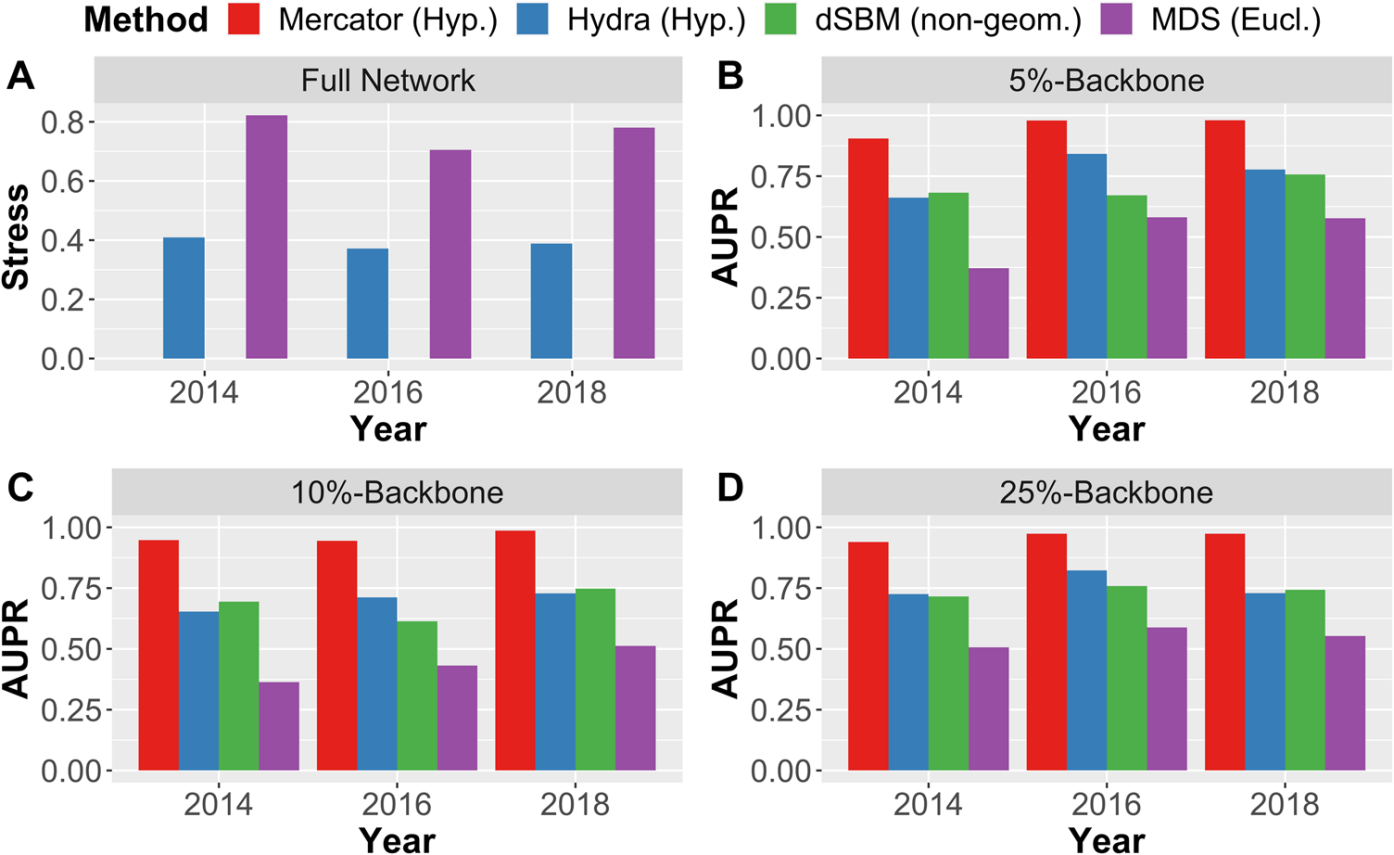
Panel (**A**): Stress (i.e. root mean square error) of network embeddings produced by hydra+ (hyperbolic target space) and multidimensional scaling/MDS (Euclidean target space). Lower stress values indicate better embedding quality. Panels (**B**)–(**D**): Area-under-Precision-Recall-curve (AUPR) for the task of reconstructing network backbones based on network representations produced by Mercator and hydra+ (hyperbolic target space), dSBM (non-geometric), and MDS (Euclidean target space). Higher AUPR values indicate better reconstruction performance.

Our next objective was to uncover the latent geometric network structure and to evaluate the suitability of a hyperbolic network model. (See “Methods” for background on hyperbolic geometry.) To this end, we applied four different network representation methods (one method embedding into two-dim. Euclidean space ${\mathbb{E}}_2$, two methods embedding into two-dim. hyperbolic space ${\mathbb{H}}_2$, and one non-geometric method) to the financial networks $N_{2014}, N_{2016}$ and $N_{2018}$ and their $p\%$-backbones for $p = 10, 25, 50$. The first two methods, multidimensional scaling^25^ (MDS) and hydra+^26,27^, calculate stress-minimizing embeddings of the weighted network distances into Euclidean and hyperbolic geometry, respectively. The third method, Mercator^28^, is a connectivity-based method (i.e. ignoring network weights) and uses a mix of machine learning and maximum likelihood estimation to infer latent coordinates in a popularity-vs-similarity-model of hyperbolic geometry; see García-Pérez et al.^28^ for details. As non-geometric representation method, we used a degree-corrected stochastic block model (dSBM)^16^, as implemented in the R-package randnet^29^, which aims to represent network structure by inferring communities and their connection probabilities; see Karrer and Newman^16^ for details. Since Mercator and dSBM are connectivity-based, they can only be meaningfully applied to the network backbones. MDS and hydra+, on the other hand, can also be applied to the full weighted networks and are directly comparable, since they minimize exactly the same objective function, but only differ in their target geometries. Figure 3 shows a comparison of the embedding quality of the different methods. For the full networks, we use stress (i.e. the root mean square error between network distances and embedded distances) as an evaluation metric, while for the backbones we use the AUPR (area under the Precision-Recall-curve) from a network reconstruction task (see “Methods” for details).

#### Comparison of methods and robustness checks

Our comparison shows that Mercator, based on hyperbolic geometry, outperforms all other methods in terms of network reconstruction performance, consistently over all three years of observation and independent of the threshold used for the extraction of the network backbone. The second hyperbolic embedding method, hydra+, yields results that are better or at least comparable to dSBM, while the Euclidean embedding method MDS performs worst. Also note that hydra+ and MDS are the only methods which can be directly applied to the full weighted network, in which case hydra+ clearly outperforms MDS in terms of embedding error.

To check the robustness of our results with respect to the method of backbone extraction, we have repeated the same analysis with backbones determined by disparity filtering^24^. The results are reported in supplementary Figure S1. While reconstruction quality deteriorates for all methods on the disparity filtered backbones, the advantage of the hyperbolic methods over the non-geometric and Euclidean methods becomes even more pronounced. Overall, we conclude that the latent geometry of the observed financial networks is—at least in low dimension—much better represented by negatively curved (hyperbolic) rather than flat (Euclidean) geometry. Moreover, the hyperbolic representations are superior even to the (non-geometric) degree-corrected stochastic block model in terms of network reconstruction quality.

#### Latent hyperbolic coordinates

**Figure 4:**
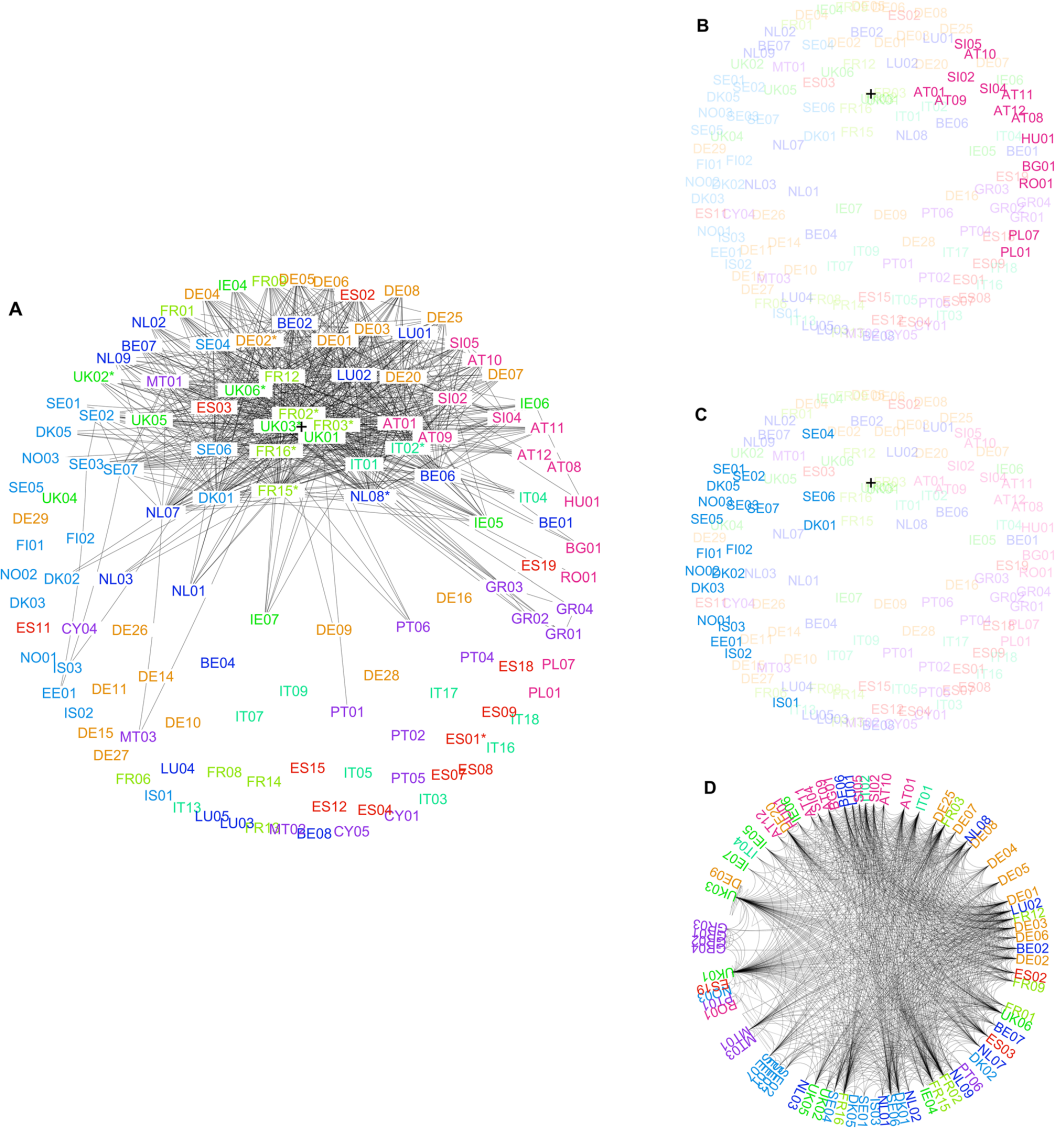
Hyperbolic Embeddings of the EBA Financial Network of 2018. Nodes are labelled by country and bank ID and coloured according to region (see Table 1 for full names). Panel (**A**) shows the full network embedding produced by the $\texttt{hydra+}$ method. Also shown is the top decile of strongest links, i.e., the connections with the largest liquidity-weighted portfolio overlap. Banks labelled as systemically important by the Financial Stability Board (G-SIBs) are indicated by asterisks. The black cross marks the capital-weighted hyperbolic center of the banking network. In panels (**B**) and (**C**) the Central/Eastern and the Nordic regional groups are highlighted to illustrate regional clustering. Panel (**D**) shows the hyperbolic embedding of the 10%-backbone of the same network, as produced by the Mercator method.

As a result of the hyperbolic embeddings we obtain for each bank node $b_i$ latent coordinates $(r_i, \theta _i)$ in the Poincaré disc model of hyperbolic space (see “Methods”). This allows us to connect the network embedding to the popularity-vs-similarity model of Papadopoulos et al.^4^ and the ${\mathbb{S}}^1$-model of Ángeles Serrano et al.^28,30^. The hyperbolic embedding of the full banking network of 2018 produced by hydra+ is shown in Fig. 4A. The embedding of the 10%-Backbone of the same network produced by Mercator is shown in Fig. 4D. Note that the hydra+-embedding attempts to give a faithful representation of all distances in the weighted network, whereas Mercator only encodes connectivity information and is harder to interpret visually. This phenomenon is exacerbated by the laws of hyperbolic geometry, in which seemingly small differences in the radial coordinate can represent large differences in hyperbolic distance. With reference to Fig. 4A, the embedded network shows a clear core-periphery structure, in line with previous studies of financial networks^31,32^ and in agreement with the pattern exhibited by the local clustering coefficient in Fig. 2.

### Structural analysis

The popularity-vs-similarity model of Papadopoulos et al.^4^ and the ${\mathbb{S}}^1$-model of Ángeles Serrano et al.^28,30^ used by Mercator offer a direct interpretation of the latent hyperbolic network coordinates in the Poincaré disc in terms of their *popularity* dimension (the radial coordinate *r*) and the *similarity* dimension (the angular coordinate $\theta$). In the context of financial networks, we hypothesized that the popularity dimension of a given bank aligns with its systemic importance, and that its similarity dimension is associated with sub-sectors of the banking system, e.g., along geographic and regional divisions. Also for the hydra+ embedding, a theoretical foundation for interpreting *r* as popularity dimension and $\theta$ as similarity dimension has been given^27^. However, due to the asymmetric distribution of banks within the Poincaré disc (Fig. 4A) for the hydra+ embedding, we calculate its geodesic polar coordinates $(r_i,\theta _i)$ with respect to the network center-of-weight, rather than the center of the Poincaré disc; see “Methods” for details (The fact that both approaches—Mercator and re-centered hydra+—lead to qualitatively very similar results can be seen as a validation of this methodology.). For the Mercator method we directly use the coordinates $(r_i,\theta _i)$ from the embedding of the 25%-backbone and perform no additional centering.

To test the first hypothesis—the association between radial coordinate *r* and systemic importance—we labelled a bank as *systemically important* in a given year, whenever it was included in the contemporaneous list of global systemically important banks (G-SIBs) as published by the Financial Stability Board^33–35^; see also Table 1. Using a Wilcoxon–Mann–Whitney test, we find a significant association between radial rank and systemic importance in all years and for both methods ($P_{2014} < .0001$, $P_{2016} < .0001$ for both methods, $P_{2018} = .0038$ for hydra and $P_{2018} = 0.0001$ for Mercator). In Table 2 we report the five top-ranked banks (most central in terms of *r*) for each year.

**Table 1:** IDs and full names of banks in the 2018 EBA Network. Banks marked by asterisk (*) were G-SIBs in all years (2014, 2016, 2018); banks marked by dagger ($\dagger$) were G-SIBs in 2014 and 2016, but not in 2018.

ID	Full name	ID	Full name
AT01	Erste Group Bank AG	GR01	Eurobank Ergasias
AT08	BAWAG Group AG	GR02	National Bank of Greece
AT09	Raiffeisen Bank International AG	GR03	Alpha Bank
AT10	Raiffeisenbankengruppe Verbund eGen	GR04	Piraeus Bank
AT11	Sberbank Europe AG	HU01	OTP Bank Ltd
AT12	Volksbanken Verbund	IE04	AIB Group plc
BE01	Belfius Banque SA	IE05	Bank of Ireland Group plc
BE02	Dexia NV	IE06	Citibank Holdings Ireland Limited
BE04	AXA Bank Europe SA	IE07	DEPFA BANK Plc
BE06	KBC Group NV	IS01	Arion banki hf
BE07	The Bank of New York Mellon SA/NV	IS02	Íslandsbanki hf.
BE08	Investar	IS03	Landsbankinn
BG01	First Investment Bank	IT01	Intesa Sanpaolo S.p.A.
CY01	Hellenic Bank Public Company Ltd	IT02	UniCredit S.p.A.*
CY04	Bank of Cyprus Holdings Public Limited Company	IT03	Banca Monte dei Paschi di Siena S.p.A.
CY05	RCB Bank Ltd	IT04	Unione Di Banche Italiane Società Cooperativa Per Azioni
DE01	NRW.Bank	IT05	Banca Carige S.P.A. - Cassa di Risparmio di Genova e Imperia
DE02	Deutsche Bank AG*	IT07	Banca Popolare Dell’Emilia Romagna - Società Cooperativa
DE03	Commerzbank AG	IT09	Banca Popolare di Sondrio
DE04	Landesbank Baden-Württemberg	IT13	Mediobanca - Banca di Credito Finanziario S.p.A.
DE05	Bayerische Landesbank	IT16	Banco BPM Gruppo Bancario
DE06	Norddeutsche Landesbank-Girozentrale	IT17	Credito Emiliano Holding SpA
DE07	Landesbank Hessen-Thüringen Girozentrale	IT18	Iccrea Banca Spa Istituto Centrale del Credito Cooperativo
DE08	DekaBank Deutsche Girozentrale	LU01	Banque et Caisse d’Epargne de l’Etat
DE09	Aareal Bank AG	LU02	Precision Capital S.A.
DE10	Deutsche Apotheker- und Ärztebank eG	LU03	J.P. Morgan Bank Luxembourg S.A.
DE11	HASPA Finanzholding	LU04	RBC Investor Services Bank S.A.
DE14	Landeskreditbank Baden-Württemberg-Förderbank	LU05	State Street Bank Luxembourg S.A.
DE15	Landwirtschaftliche Rentenbank	MT01	Bank of Valletta plc
DE16	Münchener Hypothekenbank eG	MT02	Commbank Europe Ltd
DE20	DZ Bank AG Deutsche Zentral-Genossenschaftsbank	MT03	MDB Group Limited
DE25	Deutsche Pfandbriefbank AG	NL01	Bank Nederlandse Gemeenten N.V.
DE26	Erwerbsgesellschaft der S-Finanzgruppe mbH & Co. KG	NL02	Coöperatieve Centrale Raiffeisen-Boerenleenbank B.A.
DE27	HSH Beteiligungs Management GmbH	NL03	Nederlandse Waterschapsbank N.V.
DE28	State Street Europe Holdings Germany S.à.r.l. & Co. KG	NL07	ABN AMRO Group N.V.
DE29	Volkswagen Bank GmbH	NL08	ING Groep N.V.*
DK01	Danske Bank	NL09	Volksholding B.V.
DK02	Jyske Bank	NO01	DNB Bank Group
DK03	Sydbank	NO02	SPAREBANK 1 SMN
DK05	Nykredit Realkredit	NO03	SR-bank
EE01	AS LHV Group	PL01	PKO BANK POLSKI
ES01	Banco Santander*	PL07	Bank Polska Kasa Opieki SA
ES02	Banco Bilbao Vizcaya Argentaria	PT01	Caixa Geral de Depósitos
ES03	Banco de Sabadell	PT02	Banco Comercial Português
ES04	Banco Financiero y de Ahorros	PT04	Caixa Central de Crédito Agrícola Mútuo, CRL
ES07	Caja de Ahorros y M.P. de Zaragoza	PT05	Caixa Económica Montepio Geral, Caixa Económica Bancária SA
ES08	Kutxabank	PT06	Novo Banco, SA
ES09	Liberbank	RO01	Banca Transilvania
ES11	MPCA Ronda	SE01	Nordea Bank AB (publ) †
ES12	Caja de Ahorros y Pensiones de Barcelona	SE02	Skandinaviska Enskilda Banken AB (publ) (SEB)
ES15	Bankinter	SE03	Svenska Handelsbanken AB (publ)
ES18	Abanca Holding Financiero, S.A.	SE04	Swedbank AB (publ)
ES19	Banco de Crédito Social Cooperativo, S.A.	SE05	Kommuninvest - group
FI01	OP-Pohjola Group	SE06	Länsförsäkringar Bank AB - group
FI02	Kuntarahoitus Oyj	SE07	SBAB Bank AB - group
FR01	La Banque Postale	SI02	Nova Ljubljanska banka d. d.
FR02	BNP Paribas*	SI04	Abanka d.d.
FR03	Société Générale*	SI05	Biser Topco S.à.r.l.
FR06	C.R.H. - Caisse de Refinancement de l’Habitat	UK01	Royal Bank of Scotland Group plc †
FR08	RCI Banque	UK02	HSBC Holdings plc*
FR09	Société de Financement Local	UK03	Barclays plc*
FR12	Groupe Crédit Mutuel	UK04	Lloyds Banking Group plc
FR13	Banque Centrale de Compensation (LCH Clearnet)	UK05	Nationwide Building Society
FR14	Bpifrance (Banque Publique d’Investissement)	UK06	Standard Chartered Plc*
FR15	Groupe BPCE*		
FR16	Groupe Crédit Agricole*

**Table 2:** For each year the five banks with the highest hyperbolic centrality (i.e., smallest *r* coordinate) are listed. The upper subtable corresponds to the hydra+ embedding of the full network and the lower subtable to the Mercator embedding of its 25%-backbone. Asterisks denote banks that are considered globally systemic relevant institutions (G-SIBs).

Rank (hydra+)	2014	2016	2018
1	Nordea*	BNP Paribas*	Groupe BPCE*
2	Royal Bank of Scotland*	UniCredit*	Barclays*
3	Barclays*	ING Groep*	Royal Bank of Scotland
4	Intesa Sanpaolo	Deutsche Bank*	Groupe Crédit Agricole*
5	UniCredit*	Intesa Sanpaolo	BNP Paribas*

Rank (Mercator)	2014	2016	2018
1	Deutsche Bank*	BNP Paribas*	Royal Bank of Scotland
2	BNP Paribas*	Deutsche Bank*	BNP Paribas*
3	Groupe Crédit Agricole*	HSBC*	Société Générale*
4	Commerzbank	ING Groep*	Groupe Crédit Agricole*
5	UniCredit*	UniCredit*	Barclays*

To test the second hypothesis—the association between similarity dimension $\theta$ and regional banking sub-sectors—we assigned banks to the following nine regional groups:

Spain (ES), Germany (DE), France (FR), Italy (IT), UK and Ireland (UK/IE), Nordic Region (EE/NO/SE/DK/FI/IS), Benelux Region (BE/NE/LU), Southern/Mediterranean (GR/CY/MT/PT), Central/Eastern Eur. (AT/BG/HU/LV/RO/SI).

These regions are reasonably balanced in terms of the number of banks included in the EBA panel. Using ANOVA for circular data (see “Methods”) we find a highly significant association between the angular coordinate $\theta$ and the regional group in all three years considered ($P < .0001$ in all years for both methods). This indicates that the peripheral community structure (away from the network core) of the EBA financial network is indeed strongly aligned with geographic and regional divisions in Europe. We have highlighted two different regional groups in Fig. 4B,C to illustrate the association between angular coordinate and regional structure.

### Network structure over time

The longitudinal structure of the data set allows us to track changes in the network structure over the whole time span of observations from 2014 to 2018. Note, however, that the samples of banks included by the EBA vary substantially in size and—even when restricted to the smallest sample—are not completely overlapping; see Table 3. Nevertheless, the embedding quality of the hyperbolic methods (reported in Fig. 3) is surprisingly stable over all years. This suggests that the hyperbolic model does indeed capture intrinsic qualities of the network, rather than relying on transitory structural artefacts.

**Table 3:** Sample sizes of EBA data sets

	2014	2016	2018
Number of banks (*n*)	119	51	128
Of which included in the subseq. year	43	41	

**Figure 5:**
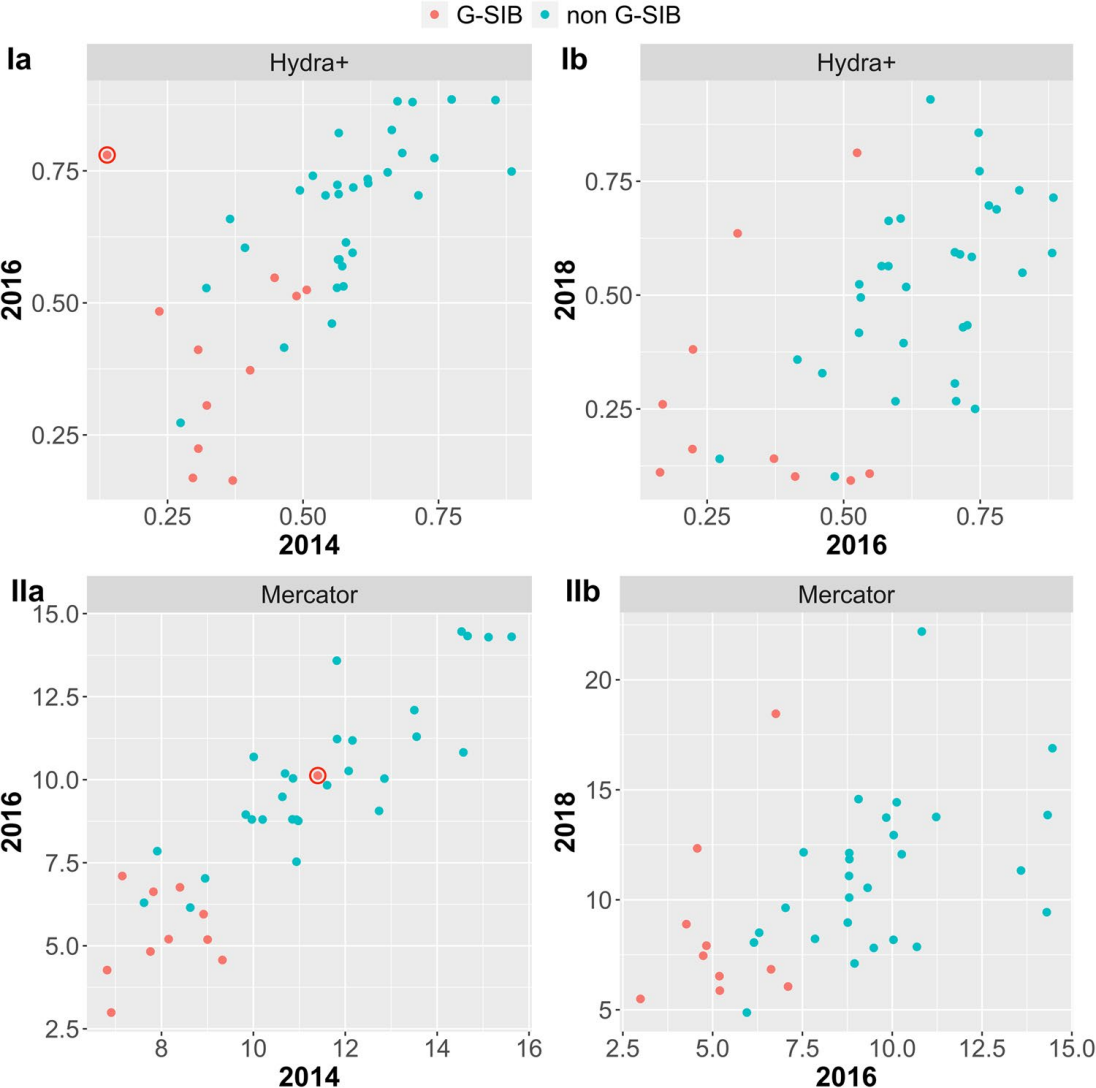
Changes in radial coordinate *r* (low values indicate high centrality) between 2014 and 2016 (**a**) and 2016 and 2018 (**b**) for the hydra+-embedding (**I**) and the Mercator-embedding (**II**). Banks considered systemically relevant (G-SIBs) at the end of the time period are marked in red. Nordea bank is circled in the panel Ia and IIa; see text for background.

We proceed to analyze the temporal changes in the latent radial coordinate *r* and angular coordinate $\theta$, corresponding to changes in systemic importance and community structure. Note that the small sample of banks included in the 2016 stress test restricts the number of banks that are included in this longitudinal analysis, cf. Table 3. The scatter plots in Fig. 5 and the corresponding Pearson’s correlations of .678 (hydra+, $P < .0001$) and .892 (Mercator, $P < .0001$) between 2014 and 2016, and .569 (hydra+, $P = .0001$) and .502 (Mercator, $P =.0015$) between 2016 and 2018 show a significant positive association between hyperbolic centrality in successive snap shots of the financial networks.

In panel Ia of Fig. 5, Nordea bank can be identified as a clear outlier, moving from a very central position in 2014 to a peripheral position in 2016. Interestingly, Nordea was one of just two banks (together with Royal Bank of Scotland) which were removed from the list of G-SIBs in the subsequent update in 2018 due to decreasing systemic importance^35^. In the Mercator embedding (panel IIa) Nordea bank does not appear as an outlier, which is likely due to the fact that some structural information is lost when the full network is reduced to its backbone.

For the angular coordinate, we account for the circular nature of the variable and compute the *circular correlation*^36^ of the angular coordinates between successive years. Only moderate associations between successive years can be observed at absolute circular correlation values of 0.211 (hydra+, $P = .1877$) and 0.01 (Mercator, $P = .9442$) between 2014 and 2016 and 0.225 (hydra+, $P = 0.1385$) and 0.383 (Mercator, $P = .0196$) between 2016 and 2018.

## Discussion

Based on data from the EBA stress tests of 2014, 2016 and the transparency exercise of 2018, we have presented strong evidence that the latent geometry of financial networks can be well-represented by geometry of negative curvature, i.e., by hyperbolic geometry. Calculating embeddings into the Poicaré disc model of hyperbolic geometry has allowed us to visualize this geometric structure and to connect it to the popularity-vs-similarity model of Papdopoulos et al.^4^ and the ${\mathbb{S}}^1$-model of Ángeles Serrano et al.^28,30^. We find that the radial coordinate (*‘popularity’*) is strongly associated with systemic importance (as assessed by the Financial Stability Board) and the angular coordinate (*‘similarity’*) with geographic and regional subdivisions. A longitudinal analysis shows that—in the observation period from 2014 to 2018—systemic importance of banks within the European banking network has stayed rather stable and has been predominated by only gradual changes. The peripheral community structure has been more variable, but has remained strongly determined by geographical divisions in all years considered.

From a broader perspective, our results indicate that hyperbolic network representations could be important tools for regulators to monitor structural change in financial networks, as they are able to distinguish changes in the systemic importance (popularity) of financial institutions from ‘peripheral changes’ (similarity) which are less relevant from a regulator’s perspective. Furthermore, our research provides an empirical basis for using hyperbolic geometry as a model space for the modelling of contagion processes and their optimal control in financial (or other) networks. Instead of modelling such processes by simulation on individual networks, a geometric model space provides the opportunity of analytic models that provide deeper insights beyond a specific case.

## Methods

### Data preparation and inference of financial networks

The financial networks were extracted from three different publicly available data sets stemming from the stress tests (in 2014 and 2016) and the EU-wide transparency exercise (in 2018) of the European Banking Authority (EBA)^14,15^. The data sets contain detailed balance sheet information from all European banks (EU incl. UK + Norway) included in the stress test/transparency exercise of the EBA in the respective year. From these data sets we extracted the portfolio values of all sovereign bonds held by the banks, split by issuing country (38 countries) and three levels of maturity (short: 0M-3M, medium: 3M-2Y, long: 2Y-10Y+), resulting in $m = 38 \times 3 = 114$ different asset classes.

For each year, this data was stored as the weighted adjacency matrix *P* (‘portfolio matrix’) of a bipartite network. The *n* rows of *P* correspond to the banks in the sample, the *m* columns to the different asset classes, and the element $P_{ik}$ to the portfolio value (in EUR) of asset *k* in the balance sheet of bank *i*. To perform a one-mode projection of this bipartite network, we followed Cont and Wagalath^23,37^ as well as Cont and Schaanning^22^: We computed the liquidity-weighted portfolio overlap (LWPO) of bank *i* and bank *j* as

1 $$\begin{aligned} L_{ij} = \sum _{k=1}^{m} \frac{P_{ik} P_{jk}}{d_k}, \end{aligned}$$

where $d_k$ is the market depth for asset *k*^22^. The LWPO measures the impact of a sudden liquidation of the portfolio of bank *i* on the portfolio value of bank *j* and vice versa. Hence, it quantifies the risk of fire-sale contagion between the banks in a financial stress scenario. The market depth of asset *k* was estimated from *P* as its total volume held by all banks in the sample, i.e., as $d_k = \sum _{i=1}^{n} P_{ik}$. Writing *D* for the diagonal matrix of market depths, (1) can be succinctly written as matrix product $L = P D^{-1} P^\top$. Finally, we set the link weight $w_{ij}$ between bank $b_i$ and $b_j$ in the one-mode projection *N* of the banking network equal to the normalized LWPO between banks $b_i$ and $b_j$, i.e., $w_{ij} := L_{ij} / \max _{i,j}L_{ij}$

### Background on hyperbolic geometry

#### The hyperboloid model

Hyperbolic geometry can be characterized as the geometry of a space of constant *negative* curvature, while the more familiar Euclidean geometry is the geometry of a flat space, i.e., a space of zero curvature. In the *hyperboloid model* of hyperbolic geometry^38,39^, *d*-dimensional hyperbolic space ${\mathbb{H}}_d$ is defined as the hyperboloid

$$\begin{aligned} {\mathbb{H}}_d = \left\{ x \in {\mathbb{R}}^{d+1}: x_0^2 - x_1^2 - \cdots - x_d^2 = 1, \; x_0 > 0\right\} \quad \text {equipped with distance} \quad {\mathrm {d}}_H(x,y) = {{\,\mathrm{arcosh}\,}}\Big (x_0 y_0 - x_1 y_1 - \cdots - x_d y_d\Big ). \end{aligned}$$

In fact, ${\mathbb{H}}_d$ endowed with the Riemannian metric tensor $ds^2 = dx_0^2 - dx_1^2 - \cdots - dx_d^2$ is a Riemannian manifold and ${\mathrm {d}}_H(x,y)$ is the corresponding Riemannian distance^38,39^. The sectional curvature of this manifold is constant and equal to $-1$. Thus, ${\mathbb{H}}_d$ is indeed a model of geometry of constant negative curvature.

#### The Poincaré disc model

While the hyperboloid model is convenient for computations, a more preferable (and popular) model for visualizations in dimension $d=2$ is the *Poincaré disc model*^38^, which also forms the basis of the popularity-vs-similarity model of Papadopoulos et al.^4^. To obtain the Poincaré disc model, the hyperboloid ${\mathbb{H}}_2$ is mapped to the open unit disc (‘Poincaré disc’) ${\mathbb{D}} = \left\{ z \in {\mathbb{R}}^{2}:{z_{1}}^{2} + {z_{2}}^{2} < 1\right\}$, parameterized by hyperbolic polar coordinates as $z_1 = \tanh (r/2) \cos \theta$, $z_2 = \tanh (r/2) \sin \theta$, using the *stereographic projection*^38^

2 $$\begin{aligned} r = \log \left( x_0 + \sqrt{x_0^2 - 1}\right) , \qquad \theta = {{\,\mathrm{atan'}\,}}(x_2,x_1), \qquad x = (x_0,x_1,x_2) \in {\mathbb{H}}_2, \end{aligned}$$

where ${{\,\mathrm{atan'}\,}}$ is the quadrant-preserving arctangent (The quadrant-preserving arctangent ${{\,\mathrm{atan'}\,}}(x_2,x_1)$, well-defined unless $x_1 = x_2 = 0$, returns the unique angle $\theta \in [0,2\pi )$ which solves $\tan \theta = x_2/x_1$ and points to the same quadrant as $(x_1,x_2)$. It is commonly implemented in scientific computing environments (e.g. in MATLAB or R) as atan2.). In the Poincaré disc model, the hyperbolic distance becomes

$$\begin{aligned} {\mathrm {d}}_B((r_1,\theta _1),(r_2,\theta _2)) = {{\,\mathrm{arcosh}\,}}\left( \cosh (r_1) \cosh (r_2) - \sinh (r_1)\sinh (r_2) \cos (\theta _1 - \theta _2)\right) \end{aligned}$$

and geodesic lines are represented by arcs of (Euclidean) circles intersected with ${\mathbb{D}}$.

### Stress-minimizing embeddings and hyperbolic centering

#### Stress-minimizing embeddings

Stress-minimizing embedding methods aim to find—for each network node $b_i$—latent coordinates $x^{i}$ in a geometric model space *G*, such that the geodesic distance between $x^{i}$ and $x^{j}$ in *G* matches—as closely as possible—a given dissimilarity measure ${\mathrm {d}}_{ij}$ (such as the weighted network distance) between nodes $b_i$ and $b_j$. This is achieved by minimizing the stress functional

3 $$\begin{aligned} {\text {Stress}}(x^{1}, \ldots , x^{n}) = {\sqrt{\frac{1}{n(n-1)}\sum _{i,j} \left( {{\mathrm {d}}_{ij}}^{\mathrm{network}} - {{\mathrm {d}}_{G}^{\mathrm{geom}}}(x^{i}, x^{j})\right) ^{2}}}, \end{aligned}$$

which measures the root mean square error between given network distances and the corresponding distances in the model space. For Euclidean geometry, this method is well-known as multidimensional scaling^25,40^, or—using a weighted stress functional—as Sammon mapping^41^. For hyperbolic space, i.e., when ${\mathrm {d}}_G^\text {geom} = {\mathrm {d}}_H$, several optimization methods for (3) have been proposed^26,27,42^. We use the hydra+ method implemented in the package hydra for the statistical computing environment R^43^.

#### Hyperbolic centering

For a point cloud $x^1, \dots , x^n$ in ${\mathbb{H}}_d$ and non-negative weights $w_1, \ldots , w_n$ summing to one, the *hyperbolic mean*^36^ or *hyperbolic center of weight*^44^ can be determined as follows: Calculate the weighted Euclidean mean ${\bar{x}} = \sum w_i x^i$, and its ‘resultant length’ $\rho = \sqrt{(\bar{x}_0)^2 - ({\bar{x}}_1)^2 - \cdots - ({\bar{x}}_d)^2}$, which is a measure of dispersion for the point cloud. The hyperbolic center *c* is then determined as $c = {\bar{x}} / \rho$ and is again an element of ${\mathbb{H}}_d$. The point cloud can be centered at *c* by transforming each point as ${\tilde{x}}^i = T_{-c} x^i$, where $T_{c}$ is the hyperbolic translation matrix (‘Lorentz boost’)

$$\begin{aligned} T_{c} = \begin{pmatrix} c_0 &{} {\bar{c}}^\top \\ {\bar{c}} &{} \sqrt{I_d + {\bar{c}} {\bar{c}}^\top }\end{pmatrix} \qquad \text {with} \qquad c=(c_0,{\bar{c}}) = (c_0, c_1, \ldots , c_d). \end{aligned}$$

In dimension $d = 2$, the stereographic projection (2) can then be applied to convert the centered coordinates ${\tilde{x}}^i$ to centered polar coordinates $(r_i, \theta _i)$ in the Poincaré disc.

### Application to financial networks

For the hydra+ embedding, the described methods were applied to the financial networks inferred from the EBA data as follows: We converted the similarity weights $w_{ij}$ (normalized LWPO) to dissimilarities ${\mathrm {d}}_{ij} = 1 - w_{ij}$. We embedded these dissimilarities by minimizing the stress functional (3), using the R-package hydra. For the resulting network embeddings, we calculated the capital-weighted network center *c* as the weighted hyperbolic mean with weights $w_i$ proportional to the total capital $\sum _{k=1}^m P_{ik}$ of bank *i* invested in all assets $a_1, \dots a_m$. After centering at the hyperbolic center *c*, we calculated the coordinates $(r_i, \theta _i)$ by the stereographic projection (2).

For the Mercator embedding and the dSBM, we first extracted network backbones, both by simple thresholding and by disparity filtering^24^. The resulting backbones were used as input for the methods provided at https://github.com/networkgeometry/mercator and the implementation of dSBM in the R-package randnet. Mercator outputs latent coordinates $(r_i, \theta _i)$ in the Poincaré disc, and the output of the dSBM method is a matrix of connection probabilities ${\hat{p}}_{ij}$ for each node pair.

For multi-dimensional scaling (MDS) the same methodology as for hydra+ was used, except that Euclidean distance (instead of hyperbolic distance) was used as ${\mathrm {d}}^\text {geom}_G$ in the objective function (3).

### Analysis of embedding results

#### AUPR and network reconstruction

To evaluate the embedding results of the network backbones, we used the following network reconstruction task: To each pair of nodes $(b_i, b_j)$, assign the score $d^\text {geom}_G(x_i,x_j)^{-1}$, where $d^\text {geom}_G$ is the geodesic distance in the geometric model space *G*, or—in case of the degree-corrected stochastic block model—the score ${\hat{p}}_{ij}$, where ${\hat{p}}_{ij}$ is the estimated connection probability between nodes *i* and *j*. Based on these scores we predict whether an edge is present between nodes $(b_i, b_j)$ or not, and construct the Precision-Recall(PR)-curve^45^ of this classifier. The area under the PR-curve (AUPR) measures the quality of this predictor, with an AUPR of 1.0 representing perfect prediction.

#### Wilcoxon–Mann–Whitney test

The Wilcoxon–Mann–Whitney^46^ test is a non-parametric test to decide whether the distributions of two populations are identical without assuming them to follow the normal distribution. Let *X* be a sample of size *m* from the first population and *Y* be a sample of size *n* from the second population. Consider the combined sample of size $m+n$ ordered from least to greatest and denote the ranks of $Y_{i}$ in this joint ordering by $S_{i}$, $i = 1, \ldots , n$. Then the test statistic $W = \sum _{i=1}^{n} S_{i}$ is the sum of the ranks assigned to the values of *Y*.

#### ANOVA for circular data

With the Analysis of Variance for circular data^36^, we test for the equality of *p* mean directions from independent circular (i.e. taking values on the unit circle) populations with von-Mises (M) distribution and the same (unknown) concentration parameter $\kappa$. We test the null hypothesis $H_{0} :\mu _{1} = \ldots = \mu _{p}$, where $\mu _{i}$ are the mean directions for the *p* populations following a $\text {M}(\mu _{i}, \kappa )$ distribution. For any circular observation $\theta$, denote $s = \sin (\theta )$, $c = \cos (\theta )$ and let ${\bar{s}}_i, {\bar{c}}_i$ be the averages within the *i*-th population. Let $n_{i}$ be the sample size, $R_{i} = \sqrt{\bar{s}_i^2 + {\bar{c}}_i^2}$ the mean resultant length of the *i*-th population, and let $n = \sum _{i=1}^{p} n_{i}$ be the size of the combined sample and *R* the overall mean resultant length based on all *n* observations. The identity

$$\begin{aligned} n-R = \left( n - \sum _{i=1}^{p} R_{i} \right) + \left( \sum _{i=1}^{p} R_{i} - R \right) = \left( \sum _{i=1}^{p} (n_{i} - R_{i}) \right) + \left( \sum _{i=1}^{p} R_{i} - R \right) \end{aligned}$$

has the approximate $\chi ^{2}$ decomposition $\chi ^{2}_{n-1} = \chi ^{2}_{n-p} + \chi ^{2}_{p-1}$^36^ and therefore, the test statistic $F = \frac{(\sum _{i=1}^{p} R_{i} - R) / (p-1)}{\sum _{i=1}^{p} (n_{i} - R_{i}) / (n-p)}$ can be derived. The null hypothesis is rejected for a given confidence level $\alpha$, when $F > F_{p-1, n-p; \alpha }$, where $F_{p-1, n-p; \alpha }$ is the $\alpha$-quantile of the *F*-distribution with $p-1$ and $n-p$ degrees of freedom^36^.

## Supplementary Information

Supplementary Information.

## Data Availability

The data analysed during the current study are available from the website of the European Banking Authority at https://www.eba.europa.eu/risk-analysis-and-data/eu-wide-stress-testing and https://eba.europa.eu/risk-analysis-and-data/eu-wide-transparency-exercise/2018.
