# Supplementary material for: The hyperbolic geometry of financial networks
Source: Sci Rep. 2021 Feb 26;11:4732. doi: 10.1038/s41598-021-83328-4 (PMC7910495; doi:10.1038/s41598-021-83328-4)
Supplement: Supplementary file 1 — Supplementary Information. [file 41598_2021_83328_MOESM1_ESM.pdf]

Supplementary Material for 'The hyperbolic geometry of financial networks'

Martin Keller-Ressel[1] and Stephanie Nargang[1]

[1]TU Dresden, Institute for Mathematical Stochastics, Dresden, 01062, Germany

Supplementary Figures

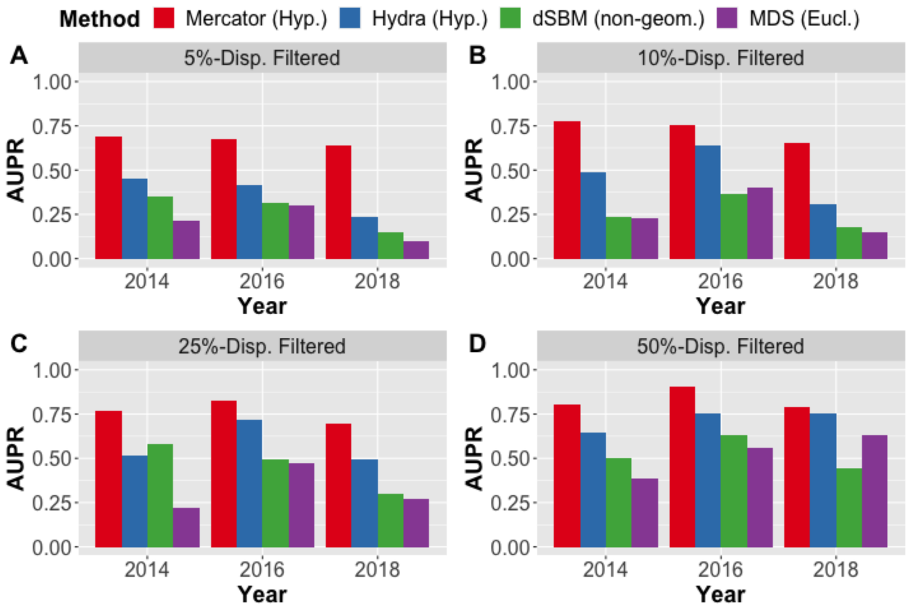

Figure S1: Area-under-Precision-Recall-curve (AUPR) for the task of reconstructing the *disparity filtered* network backbones based on network representations produced by `Mercator` and `hydra+` (hyperbolic target space), `dSBM` (non-geometric), and MDS (Euclidean target space). Higher AUPR values indicate better reconstruction performance.
